# Umbilical Cord Knots: Is the Number Related to Fetal Risk?

**DOI:** 10.3390/medicina58060703

**Published:** 2022-05-25

**Authors:** Guglielmo Stabile, Stefania Carlucci, Lucia De Bonis, Felice Sorrentino, Luigi Nappi, Giuseppe Ricci

**Affiliations:** 1 Institute for Maternal and Child Health IRCCS “Burlo Garofolo”, 34137 Trieste, Italy; giuseppe.ricci@burlo.trieste.it; 2 Department of Obstetrics and Gynecology, San Polo Hospital, Azienda Sanitaria Universitaria Giuliano-Isontina, 34128 Trieste, Italy; s.carlucci86@gmail.com; 3 Department of Medicine, Surgery and Health Sciences, University of Trieste, 34127 Trieste, Italy; lucia.debonis@burlo.trieste.it; 4 Department of Medical and Surgical Sciences, Institute of Obstetrics and Gynaecology, University of Foggia, 71121 Foggia, Italy; felice.sorrentino.1983@gmail.com (F.S.); luigi.nappi@unifg.it (L.N.)

**Keywords:** umbilical cord knot, true knot, double knot, fetal risk

## Abstract

True knots of the umbilical cord (UC) are a rare occurrence and are reported in 0.4–1.2% of deliveries. The compression of true knot of the UC can cause obstruction of the fetal circulation, leading to intra-uterine growth retardation or fetal death. Predisposing factors for the genesis of the true UC knot are numerous and include all the conditions, which lead to a relatively large uterine volume. This situation may predispose to free and excessive fetal movements. Although not all true knots lead to perinatal complications, they have been associated with adverse pregnancy outcomes, including fetal distress, fetal hypoxia, intra-uterine growth restriction (IUGR), long-term neurological damage, caesarean delivery and stillbirth. We present a rare case of operative delivery with vacuum in a multiparous woman at term of pregnancy with a double true knot of the UC. As in most cases, the diagnosis was made after delivery, as there were no fetal symptoms during pregnancy. Some authors assume that 3D power sonography may be useful in the diagnosis of true UC knots. However, 3D power Doppler cannot be considered as a definitive method. There are no specific prenatal indications to induce the physician to look for ultrasound signs suggestive of umbilical true knot. Some studies argue that cases of fetal death and fetal risk are directly related to the number of knots. We also support this thesis, even if further observational and retrospective studies are needed to demonstrate it.

## 1. Introduction

Physiologically, the umbilical cord (UC) has the role of carrying oxygen, nutrients and fluids essential for intra-uterine life.

True knots of the UC are a rare occurrence and are reported in 0.4–1.2% of deliveries [1,2]. A double true knot of the cord is a much rarer event than a single knot. The reported incidence was 0.1% [3].

It is believed that true knots are formed at 9–12 weeks of gestation when there is a relatively large quantity of amniotic fluid [1]. However, there is some evidence of knot formation also during labor [4].

The stricture of UC true knot can cause the occlusion of the fetal circulation and subsequent intra-uterine growth restriction or fetal death [1].

Predisposing factors for the formation of the true UC knot are numerous and include all the conditions, which lead to a relatively large uterine volume. This situation may predispose to free and excessive fetal movements: grand multiparous patients who have a relaxed abdominal and uterine wall, pregnancies complicated by polyhydramnios, patients with gestational diabetes who could develop polyhydramnios, chronic hypertension, small fetuses, monoamniotic twins [1,2,3].

It has been reported that this condition is more common with male fetuses, probably because, usually, the UC of male fetuses are longer than those of females [3].

The occurrence did not vary significantly with gestational age at birth [5].

There is an association between the true knot of UC and a long umbilical cord (ELUC, excessively long umbilical cord) that is well established in the literature [1,3,6]. An interesting population-based study published in 2018 [5] confirmed that the strongest risk factor for cord knot is a long umbilical cord (OR 8.42). They have built a model through which they studied the weight of each risk factor. They show, as starting with a long umbilical cord and adding other risk factors, such as parity and fetal sex, to the model, the OR did not significantly change. The risk of a cord knot was increased in polyhydramnios but adding a long cord to the model significantly reduced the effect of polyhydramnios, demonstrating the higher influence on the risk played by umbilical cord length [5]. Additionally, the effect of pre-gestational diabetes on the risk of a cord knot disappeared when a long cord was included in the model. In pregnancies after assisted reproductive techniques, they found no difference in the risk of a cord knot compared with the rest of the population. Finally, they studied the risk of recurrence demonstrating, as a cord knot recurred with an OR of 2.64 (95%CI 2.29–3.06) [5].

We present a rare case of operative delivery with vacuum in a multiparous woman at term of pregnancy with a double true knot of UC. As in most cases, the diagnosis was made after delivery, as there were no fetal symptoms during pregnancy.

## 2. Case

A 44-year-old woman with two previous spontaneous term deliveries was admitted to our center on 13 January 2021 at 40 + 3 gestational weeks due to premature rupture of the amniochorial membranes from 4 h, with clear liquid drainage.

In anamnesis, she had two previous normal pregnancies with spontaneous delivery at term, autoimmune hypothyroidism in therapy with Levothyroxine 44 gtt/day and no other comorbidities.

This was a spontaneous pregnancy and had been uneventful, and a normal quantity of amniotic fluid had been reported on an ultrasound scan with a fetus with biometrics corresponding to the time of amenorrhea. The patient had gained 10 kilos in pregnancy, and her starting body mass index was 26.9.

As for the current pregnancy, the vagino-rectal swab for GBS was positive, so that in labor, which arose and evolved spontaneously, antibiotic prophylaxis with ampicillin 2 g iv was administered as per protocol.

Labor was monitored by continuous cardiotocography. During the dilation period, the cardiotocographic trace was reactive and variable, with the fetal movements normally perceived by the patient.

With the complete dilation and valid myometrial activity, and the fetus in cephalic presentation at level 0, a pathological cardiotocographic trace appeared with multiple atypical variable decelerations. For this reason, it was decided to apply the obstetric vacuum Kiwi in the theatre. After two tractions and a slight fundic pressure, a female infant weighing 3050 g was delivered. Apgar scores were 9/10 at 1 min and 9/10 at 10 min, arterial ph 7.19–2.8 and venous ph 7.35–1.9. Active after-birth occurs with 5 IU of intravenous oxytocin. 

**Figure 1:**
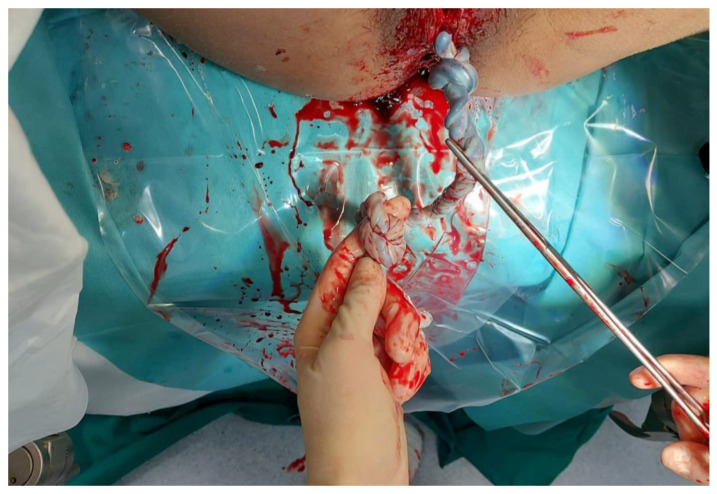
The double knot.

**Figure 2:**
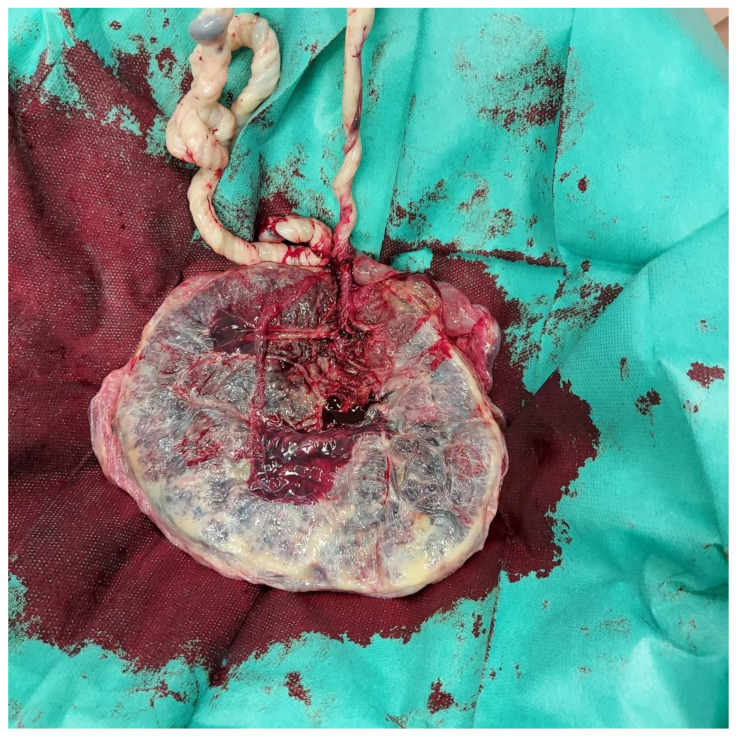
The placenta and marginal umbilical cord implant.

The UC was found to have two true knots. They were both tight and distant, respectively, 24 and 36 cm from the umbilical fetal insertion. The cord was of 60 cm, regular in length and with marginal insertion of the funiculus on the placental plate. The two umbilical arteries were present, and Wharton’s gel state was normal (Figure 1 and Figure 2). The patient suffered a vagino-perineal second-grade laceration. The total blood loss was 500cc, with no need for uterotonics in the postpartum.

The puerperium was regular. The patient and the newborn were discharged on the second day after delivery in good general conditions.

## 3. Discussion

Placental abnormalities still represent an unsolved problem responsible for a high rate of fetal compromise [7,8]. The placenta often plays a principal role, estimated at between 52% and 64%, in fetal or perinatal death, comparing different stillbirth classifications [9,10].

In particular, UC abnormalities are often associated with conditions in which fetal blood flow is reduced or interrupted as a result of alterations in the structure of the UC. 

Although not all true knots lead to perinatal complications, they have been associated with adverse pregnancy outcomes, including fetal distress, fetal hypoxia, intra-uterine growth restriction, long-term neurological damage, caesarean delivery and stillbirth [6,11,12,13]. UC true knots may lead to alterations in the cord blood flow [12]. These alterations can be caused either by direct constriction or by stretch during fetal descent, which leads to transitory cessation of cord blood flow. This condition will be expressed as non-reassuring fetal heart rate monitor [12]. A study by Carter et al. aimed to define the association between electronic fetal monitoring (EFM) and neonatal outcomes in the setting of a true knot at delivery. In this study, newborns with true knots delivered at term had similar EFM characteristics compared to neonates without true knots. Moreover, there were no significant differences in neonatal morbidity [14]. The presence of a true knot was not associated with EFM alterations, repetitive late decelerations or neonatal morbidity, confirming that a true knot can be considered a clinically benign condition [14].

Focusing on the short-term possible implications of true knots, Hershkovitz et al. [1] found a statistically significant higher rate of fetal distress and meconium-stained amniotic fluid (7% vs. 3.6%, *p* < 0.01) and a four-fold higher rate of antepartum fetal death (1.9% vs. 0.5%, *p* < 0.001) in cases of a UC true knot as compared to normal deliveries, and the caesarean section rate in these cases was significantly higher (130/841 vs. 711/68,298, *p* < 0.0001). 

A study by Kong et al. focused on clinically significant suspected fetal distress that required obstetric interventions, such as instrumental deliveries or emergency caesarean section [11]. Although the cardiac output declines during acute constriction of the umbilical vessels, the fetus can keep tissue oxygenation through its reserve if this compression is not prolonged [11]. 

In a study by Weiner et al., the OR for emergency caesarean deliveries due to non-reassuring fetal heart rate was 2.7 for the true knot and 2.9 for multiple UC loops, respectively [12]. A systematic review and meta-analysis by Hayes et al. underlined the association between the presence of true knots of the UC, stillbirth and related adverse pregnancy outcomes [13]. The probability of stillbirth was significantly higher in pregnancies with a true knot in the UC at birth than in those without, with an OR of 3.96 (95% CI 1.85, 8.47; 7 studies of 930,314 births) [13]. 

Looking instead at a possible long-term sequela of true knots, an interesting study conducted by Lichtman et al. [15], in contrast to the clear adverse impact of true knot exposure on perinatal outcome, confirmed a lack of association between true cord knots and long-term neurological morbidity (associated with hospitalization) in the offspring. 

The damage caused by the presence of the cord knot depends on the degree of venous flow obstruction, in a way that a tight knot may cause acute hypoxia, leading to immediate adverse outcome, such as intra-uterine fetal demise, while a looser knot may result in chronic mild hypoxia and a less devastating outcome. In this manner, some fetuses with knots might not be affected at all. 

To date, there are no specific prenatal indications to induce the physician to look for ultrasound signs suggestive of an umbilical true knot. Even if there is no specific sonographic feature of a true knot, some authors assume that 3D power sonography may be useful in the diagnosis of true UC knots, particularly in the third trimester of pregnancy [2,16]. Lopez et al. [17] described the prenatal diagnosis of a true knot of the umbilical cord by using the “hanging noose” sign and the analysis of the changes in the tension of the knot related to fetal movements with four-dimensional ultrasonography without a clear diagnosis in all circumstances. “Hanging noose” is a sign that is considered diagnostic of a true umbilical cord knot when a transverse section of the umbilical cord surrounded by one of its loops is observed at an ultrasound examination [17] (Table 1). However, currently, there are no clinical management guidelines. As a result, in many prenatal ultrasound units, suspected sonographic findings suggestive of a true knot of the umbilical cord are often disregarded, not documented, and patients are not aware of this potentially life-threatening condition [18,19].

## 4. Conclusions

Although umbilical cord knots are rare, when managed by a general obstetrician, these findings may be missed on ultrasound and thus not identified until birth. 

However, all of the studies mentioned above are related to singular true knots. There are no studies regarding the possible association between the number of the knots and the higher risk of fetal compromise. In conclusion, we strongly believe that the fetal risk is directly related to the number of the knots, as described by some authors [20,21]. Further observational and retrospective studies are needed to demonstrate it.

## Figures and Tables

**Table 1:** Summary from the literature.

**Risk Factors**	Major risk factors: umbilical length Minor risk factors: polyhydramnios parity fetal sex
**Diagnosis**	There are no specific prenatal indications. Some authors assume that 3D power sonography may be useful: “Hanging noose” sign
**Management**	There are no clinical management guidelines. The presence of a true knot is not associated with electronic fetal monitoring, repetitive late decelerations or neonatal morbidity but there is a statistically significant higher rate of fetal distress and meconium-stained amniotic fluid and a four-fold higher rate of antepartum fetal death. Unknown risk in case of multiple knots. The caesarean section rate in these cases was significantly higher: OR for emergency caesarean deliveries due to non-reassuring fetal heart rate was 2.7 for the true knot and 2.9 for multiple UC loops.

## Data Availability

All data are presented in the present manuscript.
